# Effect of Telerehabilitation and Outpatient Rehabilitation in Women with Breast Cancer

**DOI:** 10.3390/jfmk8030105

**Published:** 2023-07-27

**Authors:** Dalila Scaturro, Fabio Vitagliani, Maria Silvia Mangano, Sofia Tomasello, Cristiano Sconza, Stefano Respizzi, Michele Vecchio, Giulia Letizia Mauro

**Affiliations:** 1 Department of Surgical, Oncological and Stomatological Disciplines, University of Palermo, 90127 Palermo, Italy; dalila.scaturro@unipa.it (D.S.); giulia.letiziamauro@unipa.it (G.L.M.); 2 Faculty of Medicine and Surgery, University of Catania, 90121 Catania, Italy; mariasilvia.mangano@gmail.com; 3 Faculty of Medicine and Surgery, University of Palermo, 90127 Palermo, Italy; sofiatomasello@alice.it; 4 Department of Physical and Rehabilitation Medicine, Humanitas Clinical and Research Centre IRCSS, 20019 Milan, Italy; cristiano.sconza@humanitas.it (C.S.); stefano.respizzi@humanitas.it (S.R.); 5 Section of Pharmacology, Department of Biomedical and Biotechnological Sciences, University of Catania, 95124 Catania, Italy; michele.vecchio@unict.it

**Keywords:** telerehabilitation, breast cancer, disability, physical activity

## Abstract

Telemedicine was shown to be indispensable during the SARS-CoV-2 pandemic to ensure continuity of care for fragile patients. We compared a telerehabilitation program versus an outpatient rehabilitation program in women with breast cancer undergoing quadrantectomy surgery. There were 56 women with breast cancer divided into two groups: the treatment group (TG), made up of 24 patients undergoing a remote rehabilitation project program; and the control group (CG), composed of 32 patients subjected to the same rehabilitation project program in an outpatient setting. At the time of enrollment (T0) and the end of the 8 weeks of treatment (T1), the following questionnaire scores were considered: numerical rating scale (NRS), Disabilities of the Arm, Shoulder and Hand questionnaire (Quick-DASH), Piper fatigue scale (PFS)m and Breast Cancer Therapy Functional Rating Scale (FACT-B). We observed that the CG showed greater improvements than the TG in upper limb function (7.8 ± 4.2 vs. 10.9 ± 4.9; *p* < 0.05) and quality of life (27.9 ± 7.2 vs. 40.0 ± 3.3; *p* < 0.05). No difference in efficacy between the two groups was observed for pain (2.2 ± 0.6 vs. 2.3 ± 0.9; *p* = 0.64) and fatigue (3.2 ± 1.1 vs. 3.2 ± 0.6; *p* = 0.66). Telerehabilitation is a valid tool in the management of women with breast cancer in the postoperative phase. However, face-to-face rehabilitation treatment may be preferred because it is more effective as it allows the construction of a specific, personalized, and targeted rehabilitation program.

## 1. Introduction

Breast cancer is the most-frequently diagnosed cancer in women in Italy; it is estimated that there are 834,200 living people affected. According to the data of the report “The numbers of cancer in Italy 2021” in 2020, around 55,000 new diagnoses in women were reported, and in 2021, 12,500 deaths were recorded. The 5-year net survival after diagnosis is 88% [1].

Surgical treatment can range from a quadrantectomy with radiotherapy to a mastectomy. In some cases, before surgery, radiological investigations are performed to confirm that the disease is only localized in the breast and has not affected the axillary lymph node stations (lymphoscintigraphy). The subsequent surgical treatment checks the status of the lymph nodes in the ipsilateral axillary cavity and reveals the extent of the tumor and its characteristics, helping to define the therapeutic program. The most-used techniques for this purpose are sentinel lymph node biopsy (removal of only the lymph node closest to the tumor) and axillary dissection (removal of all lymph nodes) [2].

The most-frequent complications of surgery include, among others: muscle weakness, reduction of shoulder range of motion (ROM) in abduction, adduction, and flexion, joint pain, paraesthesia, and lymphedema [3]. Patients initially experience a reduction in shoulder range of motion, pain, and discomfort whenever arm movement increases tension in the axilla [4,5]. Therapeutic exercise can help reduce the extent of complications, and the patient should be encouraged to take an active part in postoperative recovery [6,7]. Indeed, patients who undertake an early rehabilitation program have a significantly better long-term outcome in terms of arm and shoulder mobility [8].

Telemedicine was shown to be an indispensable resource during the SARS-CoV-2 pandemic, above all to guarantee the continuity of care for fragile patients with multiple chronic pathologies [9,10]. Even telerehabilitation, considered a branch of telemedicine, aims to increase the accessibility of therapies and ensure therapeutic continuity for vulnerable populations, also guaranteeing savings in terms of time and resources for the health system [11,12]. However, the effectiveness of telerehabilitation is controversial [13]. Remote rehabilitation protocols have been tested, also with the help of virtual reality, during the postoperative course of patients with breast cancer, with conflicting results. Although significant improvements in global health, physical performance, and joint ROM have been observed [14,15,16], accessibility to this type of resource is limited mainly due to the complexity of the technological resources required to implement it [15]. Based on this, our research aimed to evaluate the effects of a telemedicine rehabilitation program in women with breast cancer undergoing quadrantectomy surgery, comparing it to an outpatient rehabilitation program.

## 2. Materials and Methods

### 2.1. Study Design

In our U.O.C. of Recovery and Functional Rehabilitation of the Paolo Giaccone Polyclinic in Palermo, we conducted a case–control observational study on women with breast cancer. For the data collection of this study, we used the hospital database, and we included a consecutive series of women, who in the period between January 2020 and March 2021 went to the U.O.C. of Recovery and Functional Rehabilitation of the A.O.U.P. “Paolo Giaccone” of Palermo, sent by the U.O.C. of Oncology, to undergo physiatric evaluation. The study received approval from the local ethical committee “Palermo 1” (Approval No. 03/2022) of the A.O.U.P. Paolo Giaccone of Palermo and was conducted following the Declaration of Helsinki. The processing of information and data was carried out according to the guidelines of good clinical practice (GCP).

### 2.2. Participants

The sample selection criteria used were: female gender; aged 50–60 years; history of breast cancer diagnosed for at least 24 months; quadrant surgery; no secondary localizations of active disease; no axillary lymphadenectomy; antiresorptive treatment in progress (e.g., bisphosphonates or Denosumab); ability to provide informed consent; and accessibility to electronic devices.

Patients were excluded from the sample in the case of: mastectomy surgery; active bone fractures or other musculoskeletal disorders; ongoing non-hormonal antineoplastic treatment (e.g., chemotherapy and/or radiotherapy); metastatic disease; active septic pathologies; chronic pathologies susceptible to exacerbations induced by physical exertion; altered states of consciousness; and inaccessibility to the program due to the lack of electronic devices.

### 2.3. Intervention

We recruited a total of 56 women with breast cancer, and based on the different treatments received, they were divided into two groups: the treatment group (TG), made up of 24 patients who received a remote rehabilitation project program; and the control group (CG), made up of 32 patients who received the same rehabilitation project program, but carried out on an outpatient basis.

#### 2.3.1. Treatment Group

After completing the baseline clinical assessment, participants were instructed to access the platform in person. A qualified physiotherapist himself used the digital platform Zoom (Zoom Video Communications, Inc., San Jose, CA, USA) to deliver synchronic online sessions (30–45 min each), sending each participant a link by email, half an hour before the weekly meetings. The patients were also invited to present themselves in comfortable gymnastic clothing and with tools useful for the motor program: a chair, a stick, a bottle full of water (or small weights if the patient had them), and a mat. The meetings were carried out in small groups of 4 patients three times a week, lasting 60 min and for 8 consecutive weeks, for a total of 24 sessions. The intensity and volume of physical training were established following the recommendations of the American College of Sports Medicine for cancer survivors [17]. Each session delivered online contained a battery of specific exercises divided into 3 sections: (1) warm-up, (2) resistance and balance training, and (3) cool down. The warm-up lasted 10 min and included low-intensity joint mobilization, using a stick or broomstick, and aerobic stimulation through jumping exercises and adapted jumping jacks (limiting shoulder abduction up to 90°, to prevent discomfort related to surgery or treatment). The middle part of the session lasted 40 min and involved balance exercises being performed through the single-leg stance, progressing to walking on an imaginary line. This was followed by active shoulder exercises (flexion, abduction, intra and external rotation) and resistance exercises for the core and upper and lower limbs. Dumbbells were used for resistance exercises, except for the core, pelvic lift, and split squat exercises. The exercises were alternated in segments, with 30 s of rest between exercises. The proposed resistance exercises worked both in isometric and isotonic regimes, exploiting the characteristics of each modality. The cool-down phase lasted 10 min, during which the participants performed static stretching exercises on the main muscle groups (pectoralis major, latissimus dorsi, hamstrings, adductor, and glutes) for a series of 20 s each.

#### 2.3.2. Control Group

The control group received the same rehabilitation program, but carried out on an outpatient basis, in groups of 4 people and under the supervision of an expert physiotherapist. The treatment sessions had a duration and modalities completely similar to those carried out for the telemedicine sessions.

### 2.4. Clinical Evaluation

Demographic information (age, BMI, level of education) and clinical information (side of the tumor, tumor histology, type of surgery) were retrieved from the clinical records of the recruited patients. The scores of some questionnaires were also taken into consideration, such as the numeric rating scale (NRS) [18], to evaluate the extent of pain; Disabilities of the Arm, Shoulder and Hand questionnaire (Quick-DASH) [19], to evaluate the post-surgery upper limb disability; Piper fatigue scale (PFS) [20], to evaluate the fatigue perceived by the patients; and Breast Cancer Therapy Functional Rating Scale (FACT-B) [21], to evaluate the quality of life. All this information was evaluated in two stages: at the time of recruitment (T0) and the end of the 8 weeks of treatment (T1). Upper limb disability was considered as the primary outcome. Pain, perceived fatigue, and quality of life were instead considered as secondary outcomes.

The Quick-DASH is reliable and valid for assessing upper extremity disability after breast cancer. It is an 11-item questionnaire that assesses upper limb function, its impact on daily/social activities, and the severity of upper limb symptoms. Each item is rated on a 5-point Likert scale (1 = no difficulty; 5 = not able). Participants were asked to rate the disability and severity of symptoms experienced by them while performing tasks during the past week. The total score ranges from 0 to 100, with lower scores reflecting higher disability [19]. The PFS is a validated tool for assessing cancer-related fatigue. It contains 22 items with scores ranging from 0 to 10 and includes four dimensions of subjective fatigue: behavioral/severity, affective, sensory, and cognitive/mood significance. The overall score ranges from 0 to 10, and higher scores indicate greater fatigue [20]. The FACT-B, 4th ed., includes 37 questions with answers given on a 5-point Likert scale (0 = not at all; to 4 = very much). Five dimensions of well-being are assessed: physical, social/family, emotional, functional, and additional concerns. In the present study, we used the total score, which was the sum of all dimensions, ranging from 0 to 148, with higher scores indicating better QoL. FACT-B has good reliability, validity, and internal consistency [21].

### 2.5. Statistical Analysis

Data collection was performed through the use of an electronic spreadsheet (Microsoft Excel, Version 16.58). We first calculated the sample size of the study, aiming at detecting a mean difference in the Quick-DASH (0–100) between the two groups. A power analysis was conducted with the type I error set at 0.05 and the type II error at 0.15 (85% power). The estimated sample size was 30 patients for each group to detect the least-clinically significant difference in the Quick-DASH of 7 units. The follow-up loss was estimated to be 20%. For this reason, the number of 24 patients for the treatment group and 32 patients for the control group was considered sufficient to substantiate our thesis.

Through the use of the Shapiro–Wilk test, the normality of our collected data was verified. The text and tables show continuous variables, expressed as means and standard deviations, and categorical variables, expressed as absolute numbers and percentages.

For the statistical analysis of the data, the t-test was used to compare the means between the quantitative variables, while Mood’s median test was used to compare the medians between the categorical variables. Finally, to quantify the statistical significance in the difference of the various variables examined between the two groups, we used repeated measures ANOVA. The R statistical software (R Core Team, 2021) was used to analyze the collected data. A priori results showing *p* < 0.05 were considered statistically significant.

## 3. Results

**Figure 1:**
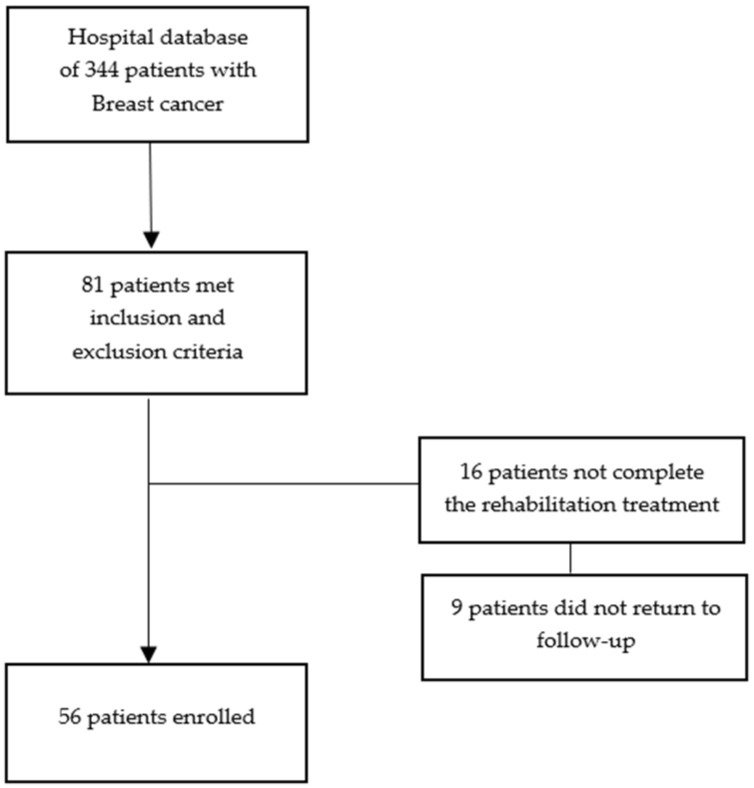
Patient recruitment.

From our hospital database comprising 344 women with breast cancer, we considered 81 women. Sixteen prematurely interrupted the rehabilitation treatment, and nine women did not show up for follow-up. A total of 56 patients were included in the study: 24 patients belonging to the treatment group and 32 patients belonging to the control group (Figure 1).

The mean age of the participants was 53.8 ± 5.25 years, and the mean BMI was 26.8 ± 4.41 kg/m^2^. The side affected by the tumor was the right in 32 patients (57.1%) and the left in the remaining 24 patients (42.9%), while the most-common tumor histotype was the lobular form, present in 30 patients (53.6%). The disability of the upper limb ipsilateral to the side of the tumor, calculated by the Quick-DASH, was 68.7 ± 7.4. The mean perceived pain was 6.5 ± 1.6. Finally, the mean score of the FACT-B scale was 104.3 ± 9.7, while that of the PFS was 7.1 ± 1.7. There were no statistically significant differences among the variables observed at baseline (Table 1).

Table 2 shows the primary and secondary outcome scores after 8 weeks of treatment in the two different groups. In the TG after 8 weeks of treatment, we observed statistically significant improvements for upper limb function (66.5 ± 5.3 vs. 74.3 ± 6.1; *p* < 0.05), for pain (6.7 ± 1.3 vs. 4.4 ± 0.7; *p* < 0.05), for quality of life (105.1 ± 6.5 vs. 77.2 ± 8.3; *p* < 0.05), and for the perception of fatigue (7.0 ± 1.2 vs. 3.8 ± 0.6; *p* < 0.05). In the CG also, after 8 weeks of treatment, we observed statistically significant improvements for all the variables examined, i.e., upper limb function (69.3 ± 7.1 vs. 80.2 ± 4.3; *p* < 0.05), pain (6.6 ± 0.9 vs. 4.3 ± 1.1; *p* < 0.05), quality of life (106.7 ± 8.1 vs. 66.7 ± 6.6; *p* < 0.05), and perceived fatigue (6.8 ± 1.4 vs. 3.7 ± 0.5; *p* < 0.05) (Table 2).

To evaluate the difference in the efficacy of the two different rehabilitation treatment regimens, we finally compared the ΔT1–T0 variations of the two groups. From this comparison, we observed that the control group showed greater improvements than the treatment group in upper limb function (7.8 ± 4.2 vs. 10.9 ± 4.9; *p* < 0.05) and quality of life (27.9 ± 7.2 vs. 40.0 ± 3.3; *p* < 0.05). No difference in efficacy between the two groups was observed for perceived pain (2.2 ± 0.6 vs. 2.3 ± 0.9; *p* = 0.64) and fatigue (3.2 ± 1.1 vs. 3.2 ± 0.6; *p* = 0.66) (Table 3).

## 4. Discussion

Numerous clinical studies present in the literature [22,23] have shown how physical training carried out during and after the surgical treatment of breast cancer can give numerous and important benefits to patients, helping to reduce the burden of the disease. Patients with BC have many problems with hand grip strength, physical activity, social interactions, and quality of life (QoL), especially after surgery [15]. Studies have shown that physical activity in these patients can be improved by rehabilitation programs and interventions [15].

During our research, we evaluated the effectiveness of an 8-week rehabilitation program carried out using telemedicine on patients undergoing quadrantectomy for breast cancer, comparing it with the same rehabilitation program carried out on an outpatient basis. Our research focused in particular on the functional capacity of the upper limb, pain, fatigue, and quality of life. Our telemedicine rehabilitation program resulted in statistically significant improvements on all the variables examined, showing overlapping efficacy with outpatient treatment as regards pain perception and fatigue. However, from the data obtained, outpatient treatment was shown to be superior in terms of efficacy as regards the recovery of the functional capacity of the upper limb ipsilateral to the surgery and for the improvement of the quality of life.

Due to the restrictions dictated by social distancing, the SARS-CoV-2 pandemic has made it necessary to research and implement new strategies for the management and treatment of different patients, including cancer patients. In this scenario, telerehabilitation has been shown to be a valid alternative, trying to remedy these needs [24,25]. The possibility of organizing meetings with the aid of digital platforms has avoided a discontinuity of assistance, allowing continuous support to our candidates [10]. The use of virtual platforms for telerehabilitation allows for easier accessibility and provides the opportunity for the patients to be reached directly at home, avoiding long journeys. Initial studies have supported the feasibility, acceptability, and effectiveness of telerehabilitation in improving participation in activities of daily living in women with breast cancer [10,12].

Additionally, other studies have supported its use in improving QoL, functional abilities, and symptom management (including those of pain, depression, anxiety, fatigue, cognitive decline, and sexual dysfunction) [26]. Lai et al. [27] found that providing telerehabilitation via the Zoom platform was similar to in-person rehabilitation in terms of the mean time required to recover baseline function. Galiano-Castillo et al. [28] used the e-CUIDATE system in the treatment of women with cancer of the senses, observing improvements in quality of life, pain, muscle strength, and fatigue. These results were maintained after the 6-month follow-up period. Their findings highlighted how telehealth approaches could be an effective alternative to traditional outpatient cancer rehabilitation. These modalities aim to minimize barriers, such as distance, time, and cost, thereby increasing accessibility for non-urban cancer survivors, facilitating rapid feedback and reducing costs [28]. A systematic review [12] regarding the use of telerehabilitation indicated the possibility of leading to similar clinical results compared to traditional rehabilitation programs, with a possible positive impact on some areas of healthcare use. Notably, recent research [24] reported a home exercise program that was shown to effectively improve symptoms of affected upper extremities (e.g., lymphedema) and led to improved QoL of breast cancer patients.

Despite the numerous new data supporting the efficacy of telerehabilitation interventions in cancer patients, the feasibility and quality of these interventions have been little explored and their efficacy remains unclear [29,30]. Our results are in line with the data present in the literature. It is known that the benefits and the impact of the effect of outpatient rehabilitation interventions on patients with breast cancer are greater than remote rehabilitation interventions [31]. This may stem from the fact that the management of chronic diseases, such as breast cancer, requires intervention in all aspects of care to achieve better health outcomes [23]. In addition to this, the main advantage of outpatient interventions is that they can carry out a daily assessment of the patient and her problems, specifically adjusting each exercise according to her needs or difficulties [23,28,31]. In this regard, Lozano-Lozano et al. [31] conducted a study to evaluate the effectiveness of a combined outpatient rehabilitation approach associated with the use of the BENECA app compared to the BENECA app alone in improving the QoL of breast cancer survivors. breast. From their data, it was seen that the combination of the BENECA app and outpatient rehabilitation significantly improved the QoL, range of motion, and upper limb function with respect to the BENECA app alone. Combining a supervised rehabilitation program with the BENECA app doubled or even tripled the clinical benefits. Tatham et al. [32] in their systematic review reported that several face-to-face interventions were effective in reducing postoperative and follow-up shoulder pain in patients with BC. Hwang et al. [33] in their randomized control trial found that pain in women with breast cancer decreased in the group receiving a supervised exercise program, while it increased in the group receiving an unsupervised independent stretching exercise program. Although the need for personalized treatments has been studied [34] and patients’ preference for face-to-face treatments has been demonstrated [35], this care is underutilized [36]. The reason for their underutilization is multifactorial and may be based on the false belief that they are only exercise or fitness programs that do not address a range of impairments that surviving cancer patients encounter [36].

However, some important limitations were present in our study. These included the retrospective nature of the study and, above all, the non-randomization of the two groups, which makes the comparability between the patients of the two groups uncertain. The fact of carrying out the rehabilitation treatment on an outpatient basis under the supervision of a physiotherapist could represent a greater motivation for the patient and could, therefore, influence the effects of the treatment itself. Furthermore, since 80% of the participants were younger than 60 years, our results may not be generalizable to older (>60 years) breast cancer patients. Finally, a further limitation is represented by the lack of a longer follow-up, which would allow us to evaluate how much these obtained benefits are maintained over time.

## 5. Conclusions

A specific and targeted rehabilitation program carried out during and after the surgical treatment of breast cancer can give numerous and important benefits to patients, helping to reduce the burden of the disease. The benefits of these rehabilitation programs in our study were observed for pain, functional capacity, fatigue, and tumor-related quality of life, although these effects were more significant in patients undergoing outpatient rehabilitation treatment. Telerehabilitation represents a valid tool available for the management of women with breast cancer in the postoperative phase, especially in those cases in which the patients have difficulty reaching the nearest hospitals. However, face-to-face rehabilitation treatment may be preferred because it is more effective as it allows the construction of a specific, personalized, and targeted rehabilitation program for the patient. RCTs should be performed in the future to compare the different efficacies of telemedicine and outpatient rehabilitation treatments.

## Figures and Tables

**Table 1:** Demographic and clinical characteristics of patients.

Characteristics	Total (*n* = 56)	Treatment Group (*n* = 24)	Control Group (*n* = 32)	*p*-Value
Age, mean ± SD	53.8 ± 5.25	54.4 ± 3.92	53.2 ± 4.31	0.86
BMI, mean ± SD	26.8 ± 4.41	27.0 ± 3.62	26.5 ± 3.91	0.77
Level education, n°(%)				0.53
Primary school	12 (21.4)	5 (20.8)	7 (21.8)	
Secondary school	28 (50.0)	13 (54.2)	15 (46.8)	
Degree	16 (28.6)	6 (25.0)	10 (31.4)	
Laterality, n°(%)				0.71
Right	32 (57.1)	14 (58.3)	18 (56.2)	
Left	24 (42.9)	10 (41.7)	14 (43.8)	
Type of breast cancer, n°(%)				0.84
Lobular	30 (53.6)	13 (54.1)	17 (53.1)	
Ductal	26 (46.4)	11 (45.9)	15 (46.9)	
Quick-DASH, mean ± SD	68.7 ± 7.4	66.5 ± 5.3	69.3 ± 7.1	0.12
NRS, mean ± SD	6.5 ± 1.6	6.7 ± 1.3	6.6 ± 0.9	0.73
FACT-B, mean ± SD	104.3 ± 9.7	105.1 ± 6.5	106.7 ± 8.1	0.43
PFS, mean ± SD	7.1 ± 1.7	7.0 ± 1.2	6.8 ± 1.4	0.57

**Table 2:** Primary and secondary outcomes after 8 weeks of treatment in the treatment group (TG) and in the control group (CG).

Characteristics	Treatment Group (*n* = 24)	Control Group (*n* = 32)	*p*-Value
Quick-DASH, mean ± SD			
T0	66.5 ± 5.3	69.3 ± 7.1	
T1	74.3 ± 6.1	80.2 ± 4.3	<0.05
*p-value*	<0.05	<0.05	
NRS, mean ± SD			
T0	6.7 ± 1.3	6.6 ± 0.9	
T1	4.4 ± 0.7	4.3 ± 1.1	0.70
*p-value*	<0.05	<0.05	
FACT-B, mean ± SD			
T0	105.1 ± 6.5	106.7 ± 8.1	
T1	77.2 ± 8.3	66.7 ± 6.6	<0.05
*p-value*			
PFS, mean ± SD			
T0	7.0 ± 1.2	6.8 ± 1.4	
T1	3.8 ± 0.6	3.7 ± 0.5	0.18
*p-value*	<0.05	<0.05	

**Table 3:** Comparison at T1 of the efficacy of the treatments in the two different groups.

Characteristics	Treatment Group (*n* = 24)	Control Group (*n* = 32)	*p*-Value
Quick-DASH, mean ± SD	7.8 ± 4.2	10.9 ± 4.9	0.01
NRS, mean ± SD	2.2 ± 0.6	2.3 ± 0.9	0.64
FACT-B, mean ± SD	27.9 ± 7.2	40.0 ± 3.3	<0.05
PFS, mean ± SD	3.2 ± 1.1	3.2 ± 0.6	0.66

## Data Availability

The data used to support the findings of this study are available from the corresponding author upon request.
